# Suicide ideation, attempt, and determinants among medical students Northwest Ethiopia: an institution-based cross-sectional study

**DOI:** 10.1186/s12991-020-00295-2

**Published:** 2020-08-09

**Authors:** Getachew Tesfaw Desalegn, Mesele Wondie, Saron Dereje, Adanech Addisu

**Affiliations:** 1 Departments of Psychiatry, College of Medicine and Health Science, University of Gondar, P. O. Box: 196, Gondar, Ethiopia; 2 University of Gondar Comprehensive Specialized Hospital, Gondar, Ethiopia

**Keywords:** Suicide ideation, Attempt, Medical students, Prevalence, Ethiopia

## Abstract

**Background:**

Suicide ideation and attempt are more highly prevalent among medical students compared to the general population. Suicidal thought negatively impacts the quality of life, physical, and mental well-being of the students. However, research into suicide ideation and attempt among medical students in low- and middle-income countries is limited. Therefore, this study aimed to explore suicide ideation and attempt and their determinants among medical students in Ethiopia have a crucial role for further intervention.

**Methods:**

An institution-based cross-sectional study was conducted on 393 medical students from March to June 2019 at the University of Gondar (UoG) in Ethiopia. Simple random sampling technique was used to select study participants. Suicide ideation and attempt were assessed using the World Health Organization Composite International Diagnostic Interview (CIDI) to assess lifetime suicide ideation and attempts during medical school. Data were collected using a self-administered questionnaire. Binary logistic regression analyses were used to identify factors associated with suicide ideation and attempt. An odds ratio (OR) with a 95% confidence interval (CI) was computed to assess the strength of the association.

**Results:**

A total of 393 participants took part with a response rate of 92.9%. The prevalence of suicide ideation and attempt among study participants was found to be 14% and 7.4%, respectively, with 95% CI (10.9–18.1) and (5.1–10.2). Being female (AOR: 5.21, 95% CI 3.42–7.20), co-morbid depression symptoms (AOR: 10.12, 95% CI 6.80–15.52), current khat chewing (AOR: 4.46, 95% CI 3.32–10.02), and poor social support (AOR: 4.46, 95% CI 3.43–9.87) were factors significantly associated with suicide ideation; whereas, female sex (AOR: 8.08, 95% CI 6.04–12.39), depression (AOR: 10.66, 95% CI 8.01–19.01**)** and history of mental illness (AOR: 5.53, 95% CI 5.20–15.50) were factors significantly associated with suicide attempt.

**Conclusions:**

In the current study, the prevalence of suicide ideation among medical students was low compared to other studies, but the suicide attempt was high. Ministry of Health should develop a guideline on how to screen and manage suicide ideation and attempt among medical students.

## Introduction

Suicide is the act of deliberately causing one’s own death [1]. Suicide ideation involves thoughts of serving as the agent of one’s own death, while a suicide attempt is a self-injurious behavior with a non-fatal outcome accompanied by implicit evidence that the person intended to die [2, 3].

Globally, the rate of suicide has increased by 60% in the past 45 years, and about one million people die from suicide every year. It is the second leading cause of death among young people, and it is higher among males than females. Suicide acts result from social, cultural, psychological, biological, and environmental factors that can interact to lead to suicidal behavior [4, 5].

Suicide ideation, attempt, and psychological distress are common among university students [6–10]. In one study, the global prevalence of suicide ideation and attempts among 36 college students was 23.3% and 3.2%, respectively [7]. Physicians are one of the highest risk groups for suicide and this problem starts during medical school [2, 11–14]. Mental distress among medical students is widespread [15] and more highly affects medical students compared with the general population [16, 17]. Suicidal behavior is a mental health problem affecting a remarkable proportion of medical students [18]. Most students experience a greater risk of suicide ideation and thoughts of dropping out of medical school [19]. The magnitude of suicide ideation among medical students in 13 Western and non-Western countries ranged from 1.8% to 53.6% [20]. The prevalence of suicide ideation and attempt among medical students in South Africa was 32.3% and 3.2%, respectively [21]. In Ethiopia, the prevalence of suicide ideation among university students was 19.9%, but there is no study regarding suicide attempt [13].

Suicide ideation and depression are strong predictors of attempted suicide among medical students [4, 17]. The rate of suicide ideation and attempt among female medical students was higher than male medical students [5, 15]. Different factors can play a role in the development of suicidal behavior. These include depression, burnout, sleep disorders, a family history of mental illnesses, previous psychiatric disorders, the number of years of study, gender, substance use, poor social support, living alone, feeling neglected by parents, having lost something valuable, the breaking of a steady love relationship and poor physical health [13, 16, 18, 20–22]. The impact of suicidal behavior among medical students includes substance use, unhealthy peer relationships, fixation on death or violence, poor academic performance, dropping out, and suicide attempt [23–25].

Even though suicidal behavior has a high prevalence among medical students, most available research evidence comes from developed countries, and little attention is paid to lower-middle-income and lower-income countries [26]. To the best of our knowledge, there has been no published study on suicide ideation, attempt, and associated factors among medical students in Ethiopia. Therefore, this study aimed to investigate the magnitude and determinants of suicide ideation and attempts among medical students in Ethiopia.

## Methods

### Study design and period

An institution-based cross-sectional study was conducted at the UoG College of Medicine and Health Sciences, March 2019, in Northwest Ethiopia.

### Study setting

The UoG is one of the oldest universities in Ethiopia. It was established in 1954 as the Public Health College and Training Center. It has 12 departments, including schools of medicine. The study was conducted on 1397 medical students. The UoG, CHMS is in Gondar town, which is 728 km from the capital city of Addis Ababa.

### Study population

Undergraduate medical students at the UoG College of Medicine and Health Sciences were included in the sample.

Sample size calculation and sampling procedures: the sample size was calculated using the single population proportion formula with a 95% CI, a 5% margin of error, and suicide ideation, and attempt of 50%, because of no published work in our country. Assuming a 10% non-response rate, 423 students were recruited randomly using stratified random sampling to allocate sample of students in each year (1 to 5), and we employed the simple random sampling technique to select each study participant.

### Data collection tools

Data were collected using a self-administered questionnaire which contained suicide ideation and attempt as the dependent variable and several other explanatory variables that included socio-demographic factors, social support, clinical factors, and substance use factors. Ever use of substances defined as consuming any substance at least once in his or her lifetime while current substance use defined as consuming specific substances within the last 3 months.

Data on social support were assessed by Oslo Social Support Scale [27]. It has three items Social Support Scale (OSS-3) which provided a brief measure of social support and functioning and it is considered to be one of the best predictors of mental health. It covered different fields of social support by measuring the number of the respondent feels close to the interest and concern shown by others and the ease of obtaining practical help from others. Social support was collected by the Oslo 3-item social support scale which had a 3-item questionnaire commonly used to assess social support and used in several studies in Ethiopia [16, 27]. The sum score scale ranges from 3 to 14 and had three broad categories: “poor support” 3–8, “moderate support” 9–11, and “strong support” 12–14 [28]. Depression was assessed using PHQ-9 questionnaire which has nine items and each item has four point Likert scale (0 = not at all to 3 = nearly every day). The score range is 0-27 and individuals who scored ten and above were considered as having depression?. Its score (5–9 mild depression, 10–14 moderate depression, 15–19 moderately severe depression, and 20–27 severe depression). It was validated in Ethiopian context with a sensitivity of 86% and specificity 67%, respectively [29].

Suicide ideation and attempts were measured according to the WHO questionnaire. If the respondent provided a “Yes” answers to the question, (“During medical school, have you ever seriously thought about committing or attempted suicide, respectively?” they were considered to have had suicidal ideation or attempt, respectively [30], and students were considered to have suicidal ideation and attempt intended to assess suicidal thought in medical students and previous study in Ethiopia [31].

### Data processing and analysis

Data were entered into Epi-Info version 7 software after checking for completeness and exported to SPSS version 20 for analysis. The bivariate analysis was done to see the association of each independent variable with the dependent variable. Those variables a *p*-value less than 0.2 were entered into the multivariate logistic regression model to identify the effect of each independent variable with the outcome variables. The strength of the association presented by the adjusted odds ratio with a 95% CI and a *p*-value less than 0.05 was considered statistically significant.

## Results

### Socio-demographic characteristics

A total of 393 participants took part in the study with a response rate of 92.9%. The mean age of the respondents was 22.16 (± 1.86) years. Out of the participants, 241 (61.3%) were male and 304 (77.4%) were from urban residence. Nearly three-fourths (72%) of the participants were Orthodox Christian followers, and more than half (55.5%) of students their cumulative grade point was between 2.76 and 3.5. The median monthly income of the participants was 1000 Birr and ranges from 50 to 6036 Ethiopian Birr (Table 1).

**Table 1:** Socio-demographic characteristics of respondents among medical students at University of Gondar, northwest Ethiopia, 2019 (*n* = 393)

Variables	Categories	Frequency	Percent
Sex	Male	241	61.3
	Female	152	38.7
Age	Mean	22.16	–
	SD	1.86	–
Religion	Orthodox	283	72.0
	Muslim	41	10.4
	Protestant	54	13.7
	Other^a^	15	3.8
Marital status	Married	25	6.4
	Single	368	93.6
Ethnicity	Amhara	205	52.1
	Oromo	91	23.3
	Tigrie	56	14.2
	Other^b^	41	10.4
Residency	Urban	304	77.4
	Rural	89	22.6
Year of study	1st	28	7.1
	2nd	73	18.6
	3rd	107	27.2
	4th	106	27.0
	5th	79	20.1
CGPA	2.00–2.75	59	15.0
	2.76–3.5	218	55.5
	> 3.5	116	29.5
Monthly income	50–1800	323	82.2
	> 1800	70	17.8

*CGPA* cumulative grade points average

Others^a^: Catholic, Waqefeta, Atheist

Others^b^: Gurage, Somali, Afar

### Clinical and social characteristics

Of the participants, 50 (12.7%) had a family history of mental illness, 13 (3.3%) had a history of mental illness, 33 (8.4%) had a family history of suicide attempt, and 122 (31%) had depression. Regarding social factors, 104 (26.5%), and 202 (51.4%) of the respondents had poor and moderate social supports, respectively (Table 2).

**Table 2:** Clinical and social characteristics of respondents among medical students at University of Gondar, northwest Ethiopia 2019 (*n* = 393)

Variables	Categories	Frequency	Percent
Family history of mental illness	Yes	50	12.7
	No	343	87.3
History of mental illness	Yes	13	3.3
	No	380	96.7
History of chronic illness	Yes	25	6.4
	No	368	93.6
Family history of suicide attempt	Yes	33	8.4
	No	360	91.6
Depression	No	271	69
	Yes	122	31
Social support	Poor	104	26.5
	Medium	202	51.4
	Good	87	22.1

### Substance use characteristics

At the moment, 168 (42.7%) were drinking alcohol, 51 (13%) were chewing khat, and 37 (9.4%) were taking tobacco. Of the participants, 205 (52.5%) were drinking alcohol, 66 (16.8%) were chewing khat, and 45 (11.5%) had been taking tobacco ever use, respectively (Table 3).

**Table 3:** Substance use characteristics of respondents among medical students at University of Gondar, northwest, Ethiopia 2019 (*n* = 393)

Variables	Categories	Ever use		Current use	
		Frequency	Percent	Frequency	Percent
Alcohol	No	188	47.8	225	57.3
	Yes	205	52.5	168	42.7
Khat	No	327	83.2	342	87
	Yes	66	16.8	51	13
Cigarette	No	348	88.5	356	90.6
	Yes	45	11.5	37	9.4 s
Others^a^	No	387	98.5	386	98.2
	Yes	6	1.5	7	1.8

NB: other^a^: Cannabis and Shisha

### The prevalence of suicide ideation and attempt

This study showed that the prevalence of suicide ideation and attempt of the participants were 14% and 7.4%, with a 95% CI (10.9–18.1) and (5.1–10.2), respectively (Tables 4 and 5).

**Table 4:** Bi-variable and multivariate analysis of suicidal ideation among medical students at the University of Gondar, Northwest Ethiopia, 2019 (*n* = 393)

Variables	Categories	Suicidal ideation		COR (95%, CI)	AOR (95%, CI)	*p*-value
		Yes	No			
Sex	Male	26	215	1	1	
	Female	29	123	1.95(1.10,2.46)	5.21(3.42,7.20)	0.001
Family history of mental illness	Yes	14	36	2.86(1.42,5.76)	1.88(0.80,4.41)	0.149
	No	41	302	1	1	
History of mental illness	Yes	4	9	2.87(0.85,9.66)	2.40(0.57,10.15)	0.23
	No	51	329	1	1	
Depression	Yes	40	82	8.32(4.38,11.84)	10.12(6.80, 15.52)	0.001
	No	15	256	1	1	
Current alcohol	Yes	32	136	2.07(1.16,3.68)	1.57(0.76,3.24)	0.39
	No	23	202	1	1	
Current khat	Yes	16	35	3.55(1.80,7.00)	4.46(3.32, 10.02)	0.01
	No	39	303	1	1	
Current cigarette	Yes	10	27	2.56(1.16,5.64)	0.53(0.13, 2.19)	0.39
	No	45	311	1	1	
Social support	Poor	25	79	5.20(1.89,7.23)	4.46(3.43, 9.87)	0.01
	Moderate	25	177	2.31(0.86,6.27)	1.95(0.67, 5.81)	0.22
	Good	5	82	1	1	

### Factors associated with suicidal ideation and attempt

Among all independent variables, female sex, depression, current and ever chewing khat, current drinking alcohol, and smoking cigarettes, history of mental illness, family history of mental illness, and social support had less than 0.2 ***p*-v**alues in the bivariate logistic regression and considered as the multivariate logistic regression model. The multivariate analysis suggested that female sex was more than five times (95%; CI 3.42**–**7.20) more likely to have suicide ideation compared with the male sex. Co-morbid depression was tenfold (95%; CI 6.80**–**15.52) more risky to develop suicide ideation compared to their counterparts. At the moment, chewing khat was 4.46 times (95%; CI 3.32**–**10.02) more risky for suicide ideation compared to students who did not chew khat and having poor social support more than four times (95%; CI 3.43**–**9.87) to develop suicide ideation compared to good social support (Table 4). On the other hand, female sex was more than eight times (95%; CI 6.04, 12.39) to develop suicide attempt compared with counterparts and depression also has 10.66 times (95%; CI 8.01, 19.01) higher risk to develop suicide attempt compared to students who did not have depression. Finally, having a history of mental illness was about 5.53 times (95%; CI 5.20, 15.50) to increase suicide attempt compared to counterparts (Table 5).

**Table 5:** Bi-variable and multivariate analysis of suicide attempts among medical students at University of Gondar, Northwest Ethiopia, 2019 (*n* = 393)

Variables	Categories	Suicide attempts		COR(95%, CI)	AOR(95%, CI)	*p*-value
		Yes	No			
Sex	Male	10	231	1	1	
	Female	19	133	3.30(1.49,6.30)	8.08(6.04,12.39)	0.001
Family history of mental illness	Yes	7	43	2.38(0.96,5.89)	1.58(0.54,4.61)	0.40
	No	22	321	1	1	
History of mental illness	Yes	4	9	6.31(1.81,8.93)	5.53(5.20,15.50)	0.02
	No	25	355	1		
Depression	Yes	21	101	6.83(2.93,12.92)	10.66(8.01, 19.01)	0.001
	No	8	263	1	1	
Ever alcohol use	Yes	20	185	2.15(0.95,4.84)	0.89(0.16, 4.96)	0.89
	No	9	179	1	1	
Ever khat use	Yes	9	57	2.42(1.05,5.60)	0.68(0.03,7.91)	0.80
	No	20	307	1	1	
Ever cigarette use	Yes	7	38	2.73(1.09,6.81)	1.37(0.07,15.86)	0.83
	No	22	326	1	1	
Current alcohol use	Yes	18	150	2.33(1.07,5.09)	2.39(0.42, 11.35)	0.32
	No	11	214	1	1	
Current khat use	Yes	8	43	2.84(1.19,6.81)	2.20(0.09,10.70)	0.84
	No	21	321	1	1	
Current cigarette use	Yes	6	31	2.80(1.06,7.40)	1.45(.06,9.00)	0.82
	No	23	333	1	1	

## Discussion

In the current study, the magnitude of suicide ideation, attempt, and their possible association with various factors were assessed among medical students in Ethiopia for the first time. The prevalence of suicide ideation and attempt during their medical education was found to be 14% and 7.4%, respectively. Regarding the prevalence of suicide ideation, our result is consistent with those of reported magnitude in other studies carried out among medical students in Taiwan, China, Australia, and Turkey, the prevalence estimated at 11.5%, 17.7%, 11.3%, 12%, respectively [22, 32, 33].

On the other hand, this finding is higher than a study done in China, the magnitude reported at 7.5% [17]. The variation may be because of instrumental, for instance, in China, suicide ideation among medical students has been assessed item nine of the patient health questionnaire the scale using the phrase “thoughts that would be better off dead or hurting yourself”. The prevalence of suicide ideation in the current study is lower than the study done among medical students in South Africa was 32.3% [21], in India, by using “Do you have thought suicide/death?” was 53.6% [34], in Turkey and Austria, students were asked five questions with dichotomous(yes/no) response format related to ever suicidal behavior were 27.3% and 37.8%, respectively [33], and in the USA, the impact of medical school on student mental health by using three suicide questions from the inventory developed by Meehan was 29.9% [35]. The variation may be due to distinctions in study designs, sample size, and the socio-cultural variations between Ethiopia, and the other countries. Moreover, the difference was suicide ideation and reactions to suicide among medical students were different in different cultural [31].

Regarding factors associated with suicide ideation, female sex had five times more risk of suicide ideation compared with male medical students. This was supported by a study carried out in China. Female medical students had higher suicidal thoughts than male medical students [10], in Pakistan female medical students a greater risk of suicide ideation than male students [5], and another meta-analysis was done among medical students and medical professionals, psychological distress was higher for female physicians and medical students compared with males [15]. Female physicians have a greater risk of suicide than other women [2]. Females attempt suicide or suicidal thoughts three times as often as males, even if this disparity remains unclear. Women have a greater vulnerability to psychosocial distress because of various hypotheses like hormonal differences, psychosocial stresses, and behavioral model of learning helplessness [1]. Socio-cultural variation between Ethiopia and others countries might be one of the reasons. For example, in Ethiopia female sex experiencing some form of trauma during their lives like gender violence, discrimination, and sexual abuse among teenagers and young adults with challenging attitudes and norms related to gender. And our culture allows men control over women. Many work with male peer groups, acknowledging young adults the strong influence that young adults can have on each others’ behavior. This may lead to high stress, social withdrawal, low self-esteem, and suicidal behavior among female.

Having depression was found to be significantly associated with suicide ideation. The odds of suicide ideation were ten times higher among students who had depression than who had no depression. Our results are consistent with findings reported in other studies, having depression among medical students was the most common predictor of suicide ideation [11, 14].

In this study, current khat chewing was 4.46 times more risky for the development of suicide ideation compared to students who had no suicide ideation. This was supported by other studies done among the university students of Ethiopia; current khat chewing was nearly two times increased suicide [13], having drug misuse among medical students was a strong predictor of suicidal thoughts [20]. Suicide risks among those who abuse substances were high in different studies [2]. Different studies have found an increased high suicidal rate among those who abuse substances. One of the main reasons why students chew khat is studying for examinations and its long-term effects are aggression, impulsiveness, cognitive impairment, depression, and suicide ideation or attempt. So khat chewing is one of the predictors of suicidal behavior.

Poor social support increased more than four times among medical students compared to their counterparts in this study. This is in line with a study done in Ethiopia [13]. Lack of social support was two times the risk of suicide ideation among the University students in Ethiopia compared to students who had moderate or good social support. Suicide ideation was high among people who had less social support from their family, friends, and other relatives [36].

On the other hand, our result of suicide attempt was in line with those of other studies conducted among medical students. For example, a study in South Africa medical students reported magnitude was 6.2% [21] and a cross-cultural investigation among similar study participants in Turkey the prevalence of suicide attempt estimated at 6.4% [33]. Similarly, the prevalence of suicide attempt among study participants was higher than those of other studies conducted: in the United Arab Emirates 1.8% [32]; in Germany, suicide attempt among medical students and young doctors was assessed by using one question from patient health questionnaire nine was 1.4% [32]; in Delhi, 2.6% had attempted to commit suicide at least once in their life [33], and in China the pooled prevalence of suicide attempt was 2.7% [9].

Our study revealed that the female sex was eightfold of attempted suicide among medical students compared to their counterparts. This is supported by a study done in Nepal; girls were nearly twofold more likely to attempt suicide compared to boys [37]. There were different studies documented in Uganda, South Africa, and Iran; females were more at risk to have a suicide attempt compared to males [38–40]. Women attempt suicide or have suicidal thoughts threefold as often as males [2].

In this study, depression and history of mental illness were strong predictors for suicide attempt. Depression is more than ten times risky for suicide attempt compared with those who had no depression and history of mental disorders was more than fivefold of suicide attempt compared to students who had no history of mental illness. These agreed with the previous studies done in South Africa [18, 21]. The previous diagnoses of depression or psychiatric disorders had strong correlations with suicide attempt [21] and suicidal behavior or attempt was higher in medical students who had depression [12]. Depression disorders account for 80% of suicide attempt while previous psychiatric disorders risk for suicide is 3 to 12 times that of non-psychiatric patients, but the degree of risk varies depending on sex, age, diagnosis, and treatment [2]. Since the questionnaire is self-administered, the participants may not give a genuine response. Cross-sectional study design cannot permit conclusions for some variables, for example, to decide whether suicidal ideation and/or attempt are risks for or consequence. This study also did not include the sixth year medical students, so this is not representative of all medical students. This finding is likely only to hint at the complex interactions between suicide ideation and attempt with explanatory variables (risk factors). Suicide ideation and/or attempt are one of components of depression diagnosis among nine items of depression symptoms so it might lead to a diagnosis bias of this outcome. Moreover, this study was on self-reported data, which might reduce objectivity and introduced the possibility of reporting bias. This study, conducted in one university in Ethiopia, which confines generalized to other settings, and finally, the use of retrospective items in the questionnaire may have incurred recall bias.

## Conclusions

The prevalence of suicide ideation among medical students was low compared to other studies, but the suicide attempt was high. Female sex, depression, current khat chewing, and poor social support were factors significantly associated with suicide ideation while female sex, depression, and history of mental illness were factors significantly associated with suicide attempts. This finding showed that suicide ideation and attempt in medical students remain a significant concern. Ministry of Health should develop a guideline on how to screen and manage suicide ideation and attempt among medical students. Future researchers should focus on preventive and treatment programs targeting the identified factors associated with suicide ideation and attempt in medical students should be conducted to strengthen and broaden these findings.

## Data Availability

The dataset during and/or analyzed during the current study is available from the corresponding author on reasonable requests.

## Abbreviations

CI: Confidence interval
CMHS: College of Medicine and Health Science
Epi-info: Epidemiological information
QOL: Quality of life
OR: Odds ratio
SPSS: Statistical Package for Social Sciences
UAE: United Arab Emirates
UOG: University of Gondar
USA: United States of America
